# Splenic Infarct Due to a Patent Foramen Ovale and Paradoxical Emboli Post-COVID-19 Infection: A Case Study

**DOI:** 10.7759/cureus.14887

**Published:** 2021-05-07

**Authors:** Emma J Norton, Nadim Sheikh

**Affiliations:** 1 Gastroenterology Department, West Suffolk NHS Foundation Trust, Bury St Edmunds, GBR; 2 Division of Anaesthesia, University of Cambridge, Cambridge, GBR

**Keywords:** covid-19, splenic infarct, patent foramen ovale

## Abstract

Hypercoagulability is now a recognized complication of COVID-19 infection. Despite this, splenic infarction remains rare and is often found incidentally, radiologically, or at autopsy. We report a case of symptomatic splenic infarction with superimposed infection, secondary to COVID-19-induced hypercoagulability in a young patient with paradoxical emboli due to an undiagnosed patent foramen ovale (PFO). This multifactorial case should prompt a level of suspicion of the patient with unexplained abdominal pain and recent COVID-19 infection.

## Introduction

In the United Kingdom, over four million individuals have tested positive for the severe acute respiratory syndrome coronavirus 2 (SARS-CoV-2) at the time of writing [1]. The disease caused by infection with the novel coronavirus, COVID-19, is usually respiratory in nature. COVID-19 also causes an acquired hypercoagulable state that can affect multiple organ systems. Increased risk of arterial and venous thromboembolism (VTE) is now a recognized complication of infection [2] even in mild disease [3]. A higher dose of VTE prophylaxis regimes is commonly prescribed to COVID-19-positive inpatients.

We report a case of symptomatic splenic infarction with superimposed infection secondary to splenic artery thrombosis six weeks post-COVID, interestingly, in a patient with a large patent foramen ovale (PFO) that likely facilitated paradoxical emboli.

## Case presentation

A young, independent gentleman in his 30s presented to a district general hospital in the United Kingdom with a short history of feeling unwell with fever, nausea, vomiting, and abdominal pain. He also had ongoing shortness of breath since a COVID-19 infection six weeks earlier, during which he was bedbound for two weeks but did not require hospital admission. As result, diagnostic workup was not completed at the time of infection. No treatment, including anticoagulation, was prescribed. He had a history of cardiomyopathy, asthma, and tinnitus. He was not known to have any prothrombotic conditions or a history of VTE/visceral infarction. His regular medications prior to admission included irbesartan, bisoprolol, bumetanide, lansoprazole, and spironolactone. He was a nonsmoker who drank minimal alcohol.

His vital signs included temperature spikes up to 38.9°C. He remained hemodynamically stable and saturated well on room air. His chest was clear on auscultation; the upper abdomen was mildly tender, but there were no signs of peritonism. He had no signs of deep vein thrombosis. BMI was 47 kg/m^2^. ECG showed sinus rhythm with Q waves in V3 and augmented Vector Foot (aVF) with T wave inversion, in the context of cardiomyopathy. Chest x-ray showed normal cardiac and mediastinal contour. The lungs and pleural spaces were clear.

**Figure 1:**
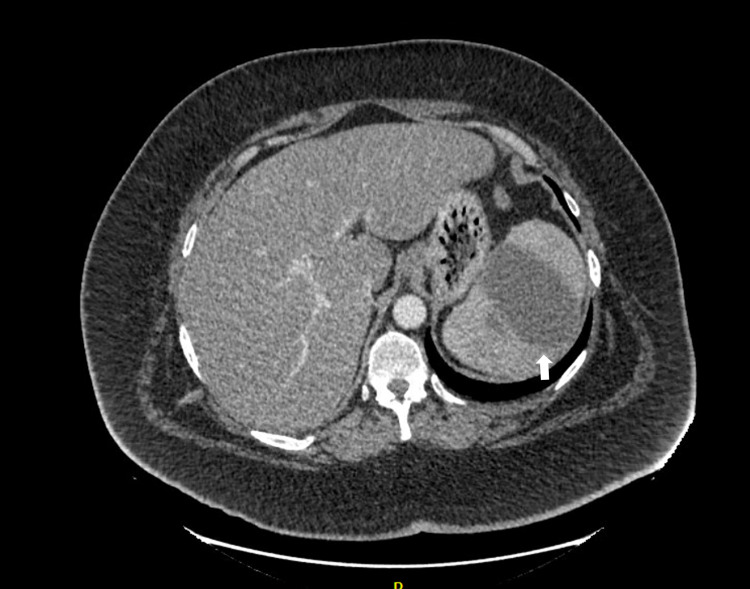
Splenic infarction (arrow) seen on arterial and PV phase CT scan PV, Portal vein; CT, computed tomography.

His inflammatory markers were raised on admission - white cell count 24.2 x10^9^/L and C-reactive protein 184 mg/L. D-dimer was 1,198 ng/ml and fibrinogen 6.40 g/L. Platelets were raised, 617 x10^9^/L, in the context of infection. Due to a high BMI, a lipid profile was checked. Total cholesterol was 4.81 mmol/L, LDL cholesterol was 2.68 mmol/L, and triglycerides were 2.90 mmol/L. Blood cultures from day zero to day one of admission were both negative. A CT pulmonary angiogram was performed to rule out pulmonary embolus (PE) because of shortness of breath and a raised D-dimer. This showed no PE but a partially imaged splenic infarct. CT abdomen demonstrated an occlusive thrombus in the splenic artery at the level of the pancreatic body. A lesion seen in the spleen was in keeping not only with infarct and liquefaction but also with superimposed infection as a possibility (Figure 1). Investigations for an underlying thrombophilia including cardiolipin antibodies, beta-2 glycoprotein, and lupus anticoagulant were advised by hematology. Ideally, investigations for a thrombotic tendency should be carried out after cessation of all anticoagulation.

Workup for a cardiac source of embolus or sepsis was performed. Transthoracic echocardiogram was of poor quality due to body habitus. Trans-esophageal echocardiogram revealed no vegetations suggestive of endocarditis, but a large PFO with a significant flow was seen across, which was confirmed with bubble study. We hypothesize that this gentleman had both hypercoagulability secondary to COVID-19 infection and prolonged bed rest as well as paradoxical emboli on a background of severe obesity causing splenic infarct.

Treatment included a course of broad-spectrum IV antibiotics (Tazocin) and anticoagulation with treatment-dose low molecular weight heparin (Tinzaparin 13,000 units subcutaneous twice daily), followed by warfarin. He was also advised on cardiovascular risk factors including weight, diet and cholesterol, and physical inactivity. Outpatient cardiology review for consideration of referral for PFO closure was arranged.

## Discussion

Splenic infarct is a rare but documented complication in COVID-19-infected patients in both clinical and autopsy studies [4-10]. Incidence in non-COVID patients is estimated at 0.016% [11]. Splenic infarct is often found incidentally and partially imaged on CT scans of the chest, as in this case. As abdominal imaging is not a commonplace in the diagnostic workup of COVID-19, a level of clinical suspicion is required for patients presenting with abdominal pain and current or previous infection [12]. The complications of splenic artery thrombosis include hemorrhage, abscess, and pseudocyst formation [13].

The most common cause of arterial thrombosis is atherosclerosis. Rarer causes of splenic infarct include cardioembolic from intracardiac thrombi, PFO with paradoxical emboli, or valvular vegetations. Hypercoagulable states (of which antiphospholipid is the most common), autoimmune disease, associated infection, and hematological disease are also known causes [11,13]. This patient likely had a multifactorial etiology due to anatomical variants (PFO) and hypercoagulability (obesity, immobility, and COVID-19 infection).

Hypercoagulability in COVID-19 infection is likely due to thrombo-inflammation, but the exact mechanism of coagulopathy remains unclarified. Similarly, it is also unknown if hemostatic changes directly result from the SARS-CoV-2 virus or the cytokine storm observed later during illness [7]. COVID-19 patients exhibit thrombocytosis, a raised D-dimer, and fibrin degradation products, which correlate with disease severity and poorer prognosis [14]. Interestingly, many are positive for lupus anticoagulant that can prolong clotting and increase thrombosis risk [15] - this is also true in patients with non-COVID-related splenic infarction. Clinical sequelae of hypercoagulability include pulmonary embolism, most commonly, in addition to ischemic stroke, limb ischemia, and visceral infarction [4]. In the COVID-19 inpatients at our trust, prophylactic low molecular weight heparin is dosed higher than that in non-COVID-19 patients and is also based on weight and D-dimer (an indicator for disease severity) [16]. Treatment of established thromboembolism related to COVID-19 requires further clarification, including anticoagulant of choice and intended duration. Studies in non-COVID patients have suggested lifelong anticoagulation in cryptogenic or visceral infarction due to a high recurrence rate [13].

## Conclusions

Splenic infarction is a rare but recognized complication of COVID-19 infection, often picked up incidentally. This unique case presents a patient with a dual mechanism for splenic artery thrombosis: a PFO with paradoxical emboli and hypercoagulability secondary to recent COVID-19 infection in an obese patient. Multifactorial etiology of visceral infarction in COVID-19 should be considered in diagnostic workup and management. Visceral infarction, particularly when driven by SARS-CoV-2 infection, requires more research into its underlying pathogenesis, investigations, and best management.
